# Apatinib-based targeted therapy against pulmonary sarcomatoid carcinoma: a case report and literature review

**DOI:** 10.18632/oncotarget.25989

**Published:** 2018-09-14

**Authors:** Xiaofeng Li, Yueming He, Jinfeng Zhu, Hongxia Pang, Yongwei Lin, Jinyang Zheng

**Affiliations:** ^1^ Department of Oncology, Affiliated Quanzhou First Hospital of Fujian Medical University, Quanzhou 362000, China; ^2^ Department of Respiratory Diseases, Affiliated Quanzhou First Hospital of Fujian Medical University, Quanzhou 362000, China; ^3^ Department of Pathology, Affiliated Quanzhou First Hospital of Fujian Medical University, Quanzhou 362000, China

**Keywords:** angiogenesis, apatinib, sarcomatoid carcinoma, targeted therapy

## Abstract

Sarcomatoid carcinoma is a rare malignancy characterized by a combination of epithelial and sarcoma or sarcoma-like components. In this study, we reported one case of pulmonary sarcomatoid carcinoma and evaluated the safety and efficacy of apatinib, a tyrosine kinase inhibitor selectively targeting vascular endothelial growth factor receptor 2, in treating this disease. The tumor mass was detected in the left lung of a 75-year-old man and showed positive immunostaining for cytokeratin (CK) 7, CK8, smooth muscle actin, CD31, and CD34. Next-generation sequencing analysis identified 4 mutations in *NF1* (p.Q347Sfs^*^29), *CDKN2A* (p.G23V), ERBB3 (p.V104L), and *TP53* (p.V157F) genes. The patient was given apatinib (250 mg) orally once per day. Sustained tumor regression was observed after apatinib treatment. There was no sever complication associated with apatinib therapy. In conclusion, apatinib-based targeted therapy may represent an important option for patients with sarcomatoid carcinoma.

## INTRODUCTION

Sarcomatoid carcinoma is a rare form of cancer characterized by a combination of epithelial and sarcoma or sarcoma-like components [1]. Sarcomatoid carcinoma has been detected at multiple anatomic sites such as the prostate [2], lung [3], gallbladder [4], jejunum [5], and pelvic cavity [6]. Pulmonary sarcomatoid carcinoma accounts for 0.1–0.4% of all pulmonary cancers [7]. It has a highly metastatic property and exhibits a poor response to conventional chemotherapy [8, 9]. Therefore, it is of significance in developing novel therapeutic strategies against this malignancy.

Apatinib is a tyrosine kinase inhibitor that selectively targets vascular endothelial growth factor receptor 2 (VEGFR2) expressed on tumor cells and shows a broad range of anticancer activity [10]. A randomized, double-blind, placebo-controlled phase III trial demonstrated that apatinib treatment resulted in a significant increase in median overall survival (6.5 *vs*. 4.7 months) and progression-free survival (2.6 *vs*. 1.8 months) in patients with chemotherapy-refractory advanced gastric cancer, compared to the placebo group [11]. A single-center randomized controlled trial showed that the combination of transcatheter arterial chemoembolization (TACE) with apatinib conferred survival benefits to patients with advanced hepatocellular carcinoma (HCC) [12]. In addition, several studies involving individual cases have documented the anticancer activity of apatinib in patients with advanced disease [13, 14].

In this report, we described one case of sarcomatoid carcinoma in the lung and showed the therapeutic effect of apatinib against this malignancy.

## CASE REPORT

**Figure 1:**
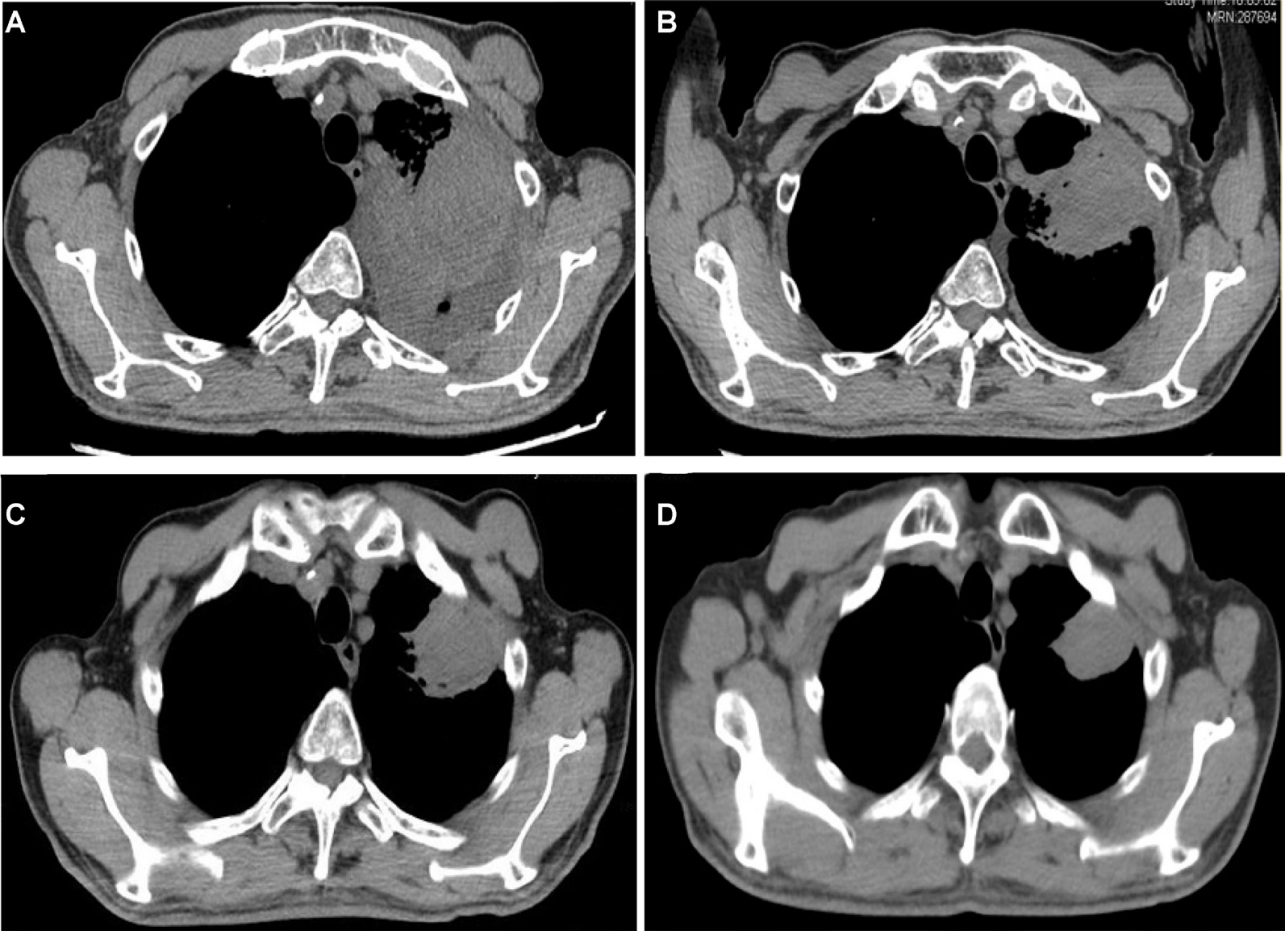
Chest CT showed tumor regression in the left lung after apatinib therapy (**A**–**D**) before and 1, 3, and 9 months after treatment, respectively.

**Figure 2:**
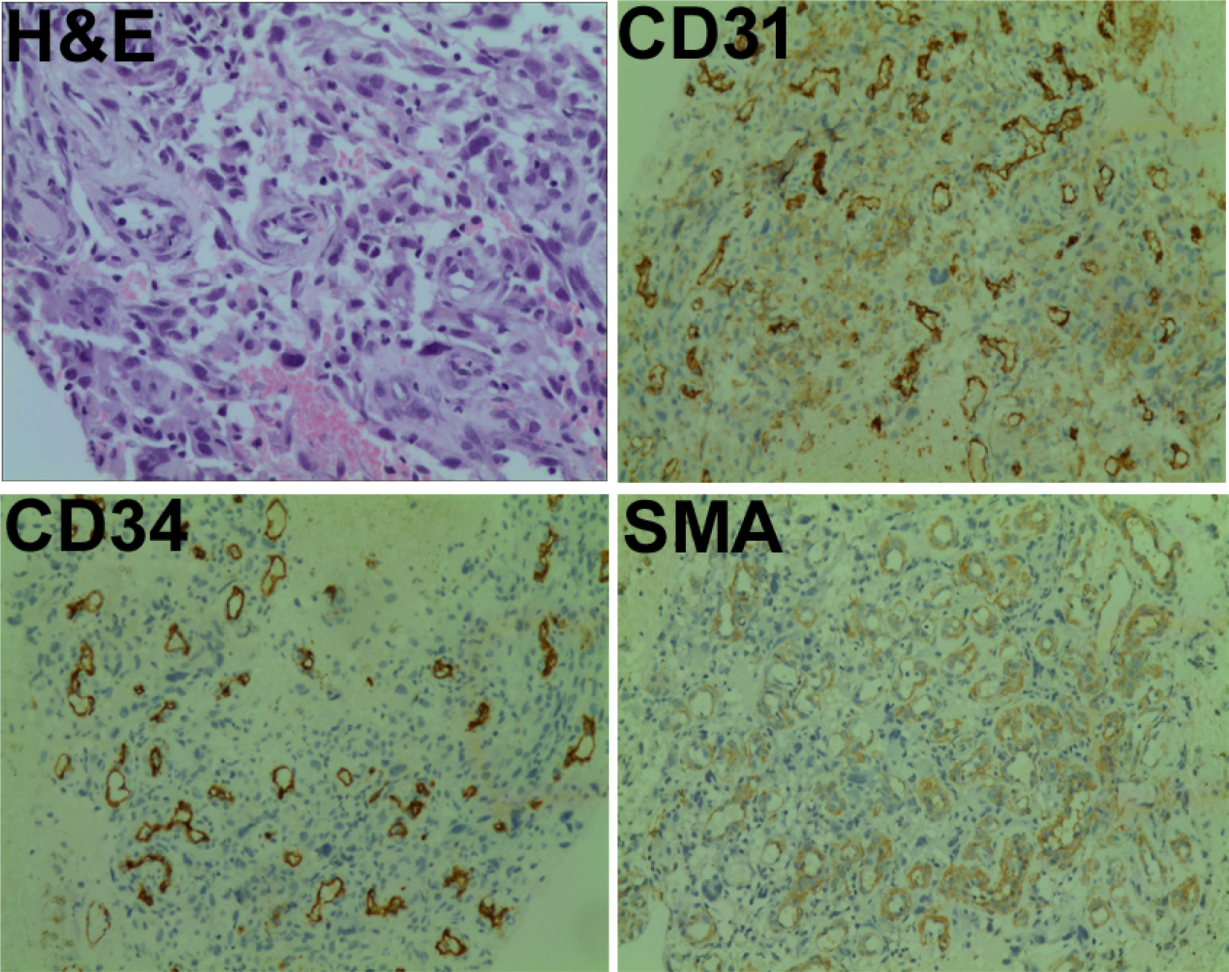
Pathological findings Tumor samples were subjected to hematoxylin-eosin (H&E) staining or immunostained for smooth muscle actin (SMA), CD31, and CD34. Magnification: ×200.

A 75-year-old man was admitted to our hospital on June 15, 2016 with a chief complaint of coughing and blood-stained sputum for over 1 month. He presented chest stuffiness and shortness of breath and required continuous oxygen inhalation. The patient also presented coronary arteriosclerosis. There were no symptoms of dizziness, chest pain, fever, and vomiting. He had a smoking history of over 60 years and smoked up to 30 cigarettes a day. Tumor markers carcinoembryonic antigen and neuron-specific enolase were within the normal range. Chest computed tomography (CT) revealed a soft mass (63 × 48 mm) and signs of pneumonia in the left lung (Figure 1A). Swollen lymph nodes above the left clavicle were evident on color Doppler ultrasound images. CT-guided lung biopsy was conducted. The mass was histologically diagnosed as sarcomatoid carcinoma, which was staged as IV according to the 8th edition of the AJCC/UICC TNM staging system for lung cancer. Supraclavicular lymph node metastasis was detected. Immunohistochemistry showed that tumor cells were positive for cytokeratin (CK) 7, CK8, and thyroid transcription factor 1 (TTF-1) and negative for CK5/6, p63, p40, CD56, and synaptophysin. Of note, the tumor was abundantly vascular, with strong immunostaining for smooth muscle actin (SMA), CD31, and CD34 (Figure 2). As determined by next-generation sequencing, this patient displayed 4 mutations in *NF1* (p.Q347Sfs^*^29), *CDKN2A* (p.G23V), *ERBB3* (p.V104L), and *TP53* (p.V157F) genes (Table 1).

**Table 1:** Detection of mutations in the patient with pulmonary sarcomatoid carcinoma by next-generation sequencing

Gene	Chromosomal location	Reference sequence	Nucleotide change	Amino acid change
*NF1*	chr17:29527590	NM_001042492	c.1039del	p.Q347Sfs^*^29
*CDKN2A*	chr9:21974759	NM_000077	c.G68T	p.G23V
*TP53*	chr17:7578461	NM_001126112	c.G469T	p.V157F
*ERBB3*	chr12:56478854	NM_001982	c.G310T	p.V104L

The patient had an ECOG performance status of 3 and thus was precluded from chemotherapy. After receiving informed consent, he was given apatinib (250 mg) orally once per day from June 27, 2016. Haemoptysis symptoms disappeared and chest stuffiness was relieved 5 days after apatinib treatment. At 10 days after treatment, he restored normal breathing. Follow-up CT at 1 (Figure 1B), 3 (Figure 1C), and 9 (Figure 1D) months showed that the tumor regressed to 54 × 43 mm, 41 × 40 mm, and 36 × 28 mm, respectively. Until drafting the manuscript (14 months after therapy), tumor regression was observed. There was no evident complication associated with apatinib therapy.

## DISCUSSION

Clinical detection of sarcomatoid carcinoma is a rare event [1–3]. In this study, we reported a case of sarcomatoid carcinoma occurring in the lung. Compared to other types of lung cancer, pulmonary sarcomatoid carcinoma has a worse overall survival [15]. Pulmonary sarcomatoid carcinoma is characterized by frequent gene mutations. Li *et al.* [3] performed next-generation sequencing in 7 patients with pulmonary sarcomatoid carcinoma and identified 136 putative somatic variants and one gene fusion. Terra *et al.* [16] analyzed 33 cases of pulmonary sarcomatoid carcinoma by next-generation sequencing and found that 72% had at least one genetic variant, 58% *TP53* mutations, and 30% *KRAS* mutations. Similarly, Lococo *et al.* [17] reported that 39 of the 49 pulmonary sarcomatoid carcinomas (80%) carried at least one mutation. Consistent with the previous studies, we also identified *TP53* mutation in this case. Of note, we found novel mutations in *NF1*, *CDKN2A*, and *ERBB3* genes. NF1 acts as a tumor suppressor and its loss-of-function mutation results in activation of the RAS/RAF/MAPK signaling pathway, consequently contributing to tumor progression [18, 19]. In this study, we showed that the mutation (c.1039del) led to the glutamine to serine change at position 347 and introduction of a stop codon, which should terminate protein translation prematurely. *CDKN2A* mutation is also linked to development of cancer [20, 21]. It has been reported that *CDKN2A* p.Arg112dup mutation carriers have an elevated risk for pancreatic, lung, head and neck and gastro-oesophageal carcinomas [21]. ERBB3 is implicated in multiple aspects of tumor biology [22, 23]. ERBB3 mutations increase the response to afatinib in platinum-refractory metastatic urothelial carcinoma [22]. Taken together, it is suggested that genetic mutations, in particular in *TP53*, *NF1*, *CDKN2A*, and *ERBB3* genes may contribute to the pathogenesis of sarcomatoid carcinoma.

Due to poor ECOG performance status, the patient reported in this study did not receive chemotherapy. CD31 and CD34 immunostaining analysis showed remarkably abundant microvessels in the tumor mass of our patient, indicating active tumor angiogenesis. A previous study has provided evidence that mutation in *NF1* gene can augment angiogenesis in a mouse model by promoting endothelial cell proliferation and migration [24]. p53 functional deficiency was reported to enhance fibroblast-mediated angiogenesis in colon cancer [25]. *TP53* mutations are associated with the sensitivity to VEGF/VEGFR inhibitors [26]. *TP53* mutational status has been identified as a predictive biomarker of response to VEGFR inhibitors in advanced sarcoma [27]. These studies suggest that the substantial tumor angiogenesis in the present case may be a result of mutations in multiple genes including *TP53* and *NF1*.

Given the presence of active tumor angiogenesis, we sought to control disease progression in the patient by targeting pro-angiogenic signaling. Apatinib, as a tyrosine kinase inhibitor targeting VEGFR2, has exhibited anticancer activity on many cancer types [10, 11, 28]. Co-administration of apatinib has been reported to augment antitumor activity of gefitinib on non-small cell lung cancer with resistance to epidermal growth factor receptor tyrosine kinase inhibitors [14]. Zhang *et al.* [29] reported that apatinib leads to partial or complete response in 2 cases of refractory recurrent malignant gliomas. Apatinib can improve progression-free survival and overall survival in patients with chemotherapy-refractory advanced metastatic gastric cancer [30]. Deng *et al.* [31] described that a case with for chemotherapy-refractory advanced epithelial ovarian cancer obtained survival benefits from apatinib treatment. In the present study, sustained apatinib treatment caused a marked tumor regression, without producing evident toxicities. The good response observed may be ascribed to suppression of tumor angiogenesis caused by mutation of specific key genes. In support of this hypothesis, it has been documented that apatinib therapy can evoke stable disease response in advanced lung adenocarcinoma patients with *KRAS* mutation [13]. However, the exact mechanism governing apatinib response needs to be further clarified.

In summary, our present case confirms frequent genetic mutations occurring in pulmonary sarcomatoid carcinoma. To our best of knowledge, this is the first report of the successful treatment of pulmonary sarcomatoid carcinoma with apatinib. Prospective studies are warranted to validate the therapeutic efficacy of apatinib in sarcomatoid carcinomas.
